# Successful surgical management of an aggressive IgG4-related sclerosing esophageal inflammatory pseudotumor: a case report and review of literature

**DOI:** 10.1186/s13019-023-02317-y

**Published:** 2023-07-04

**Authors:** Hoang Nguyen, Duc Huan Pham, Tuan Hiep Luong

**Affiliations:** 1 Department of Gastrointestinal and Hepatobiliary surgery, Hanoi Medical University Hospital, Hanoi, Vietnam; 2 Department of Surgery, Hanoi Medical University, 1st Ton That Tung Street, Dong Da, Ha Noi, Hanoi, 11521 Vietnam; 3 Center for Gastroenterology - Hepatobiliary - Urology - Vinmec Times City International Hospital, Hanoi, Vietnam; 4 Department of Gastrointestinal and Hepato-pancreato-biliary surgery, Bach Mai Hospital, Hanoi, Vietnam

**Keywords:** Inflammatory pseudotumor, Esophagus, Esophagectomy

## Abstract

**Background:**

Inflammatory pseudotumor (IPT) of the esophagus is a very rare benign lesions which clinical presentation is not clear and difficult to make a definitive diagnosis preoperatively.

**Case presentation:**

In this report, we presented a case of a 24-year-old female with signs of severe malnutrition state due to dysphagia increasing gradually and losing 10 kg in weight for 2 months. Comprehensive preoperative radiologic investigations were proceeded with a circumferential severe stricture caused smooth submucosal swelling in the esophagus under 23 cm from the upper dental arch and two times of negative biopsy. Due to the aggressive clinical symptoms and gross lesion characteristics, the patient underwent laparoscopic-thoracoscopic esophagectomy and reconstruction with a gastric tube. Histopathological examination showed that the squamous epithelium of the esophagus had a small, benign nucleus, the submucosal layer and the smooth muscle layer increased fibrous, with infiltrating many lymphocytes, plasma cells, and macrophages. Immunohistochemical staining was negative for CD68, CD34, Desmin and ALK markers, and there was an increase in the number of IgG4-positive plasma cells. The final diagnosis was an aggressive IgG4-related sclerosing esophageal inflammatory pseudotumor.

**Conclusions:**

Inflammatory pseudotumor of the esophagus is an extremely rare benign lesion but could led to aggressive clinical presentation. The gold standard of diagnosis is histopathological examination of surgically removed specimens. Radical resection is still the most efficient treatment method.

## Background

Benign lesions account for about 0.5% of the lesions of the esophagus in prevalence but may represent in up to 20% on autopsy [1, 2]. Inflammatory pseudotumor (IPT) is a very rare type of the esophageal benign lesions with an unclear pathogenesis pathology as well as clinical manifestations, that lead to the difficulty to make a definitive diagnosis preoperatively [3]. IPT is more common in the lungs but can be seen in the liver, spleen or gastrointestinal tract, but extremely rarely in the esophagus [4–7]. There are very few cases of esophageal IPT reported in English literature until now, which the first cases were described by LiVolsi et al. in 1975 [3, 8–10]. Clinically, this disease may present as a single or multiple (polypoid) mass lesions, with a pathological characteristic of multiple inflammatory cell infiltration (fibroblasts, necrotic cells, reactive granulomas, and rhabdoid fibroblasts) without presence of malignant cells [4, 11]. The origin of esophageal IPT was unknown, with hypothesis of inflammation related to trauma or surgical – endoscopic interventions [11]. Immune-autoimmune condition was found in some IPTs, with presence of IgG4-positive plasma cells, and in these cases, systemic corticosteroids therapy was considered [8, 12, 13].

We report a clinical case of IgG4-related sclerosing IPT that involves the thoracic esophagus and successfully surgical management by undergoing laparoscopic-thoracoscopic esophagectomy and reconstruction with a gastric tube.

## Case presentation

**Fig. 1:**
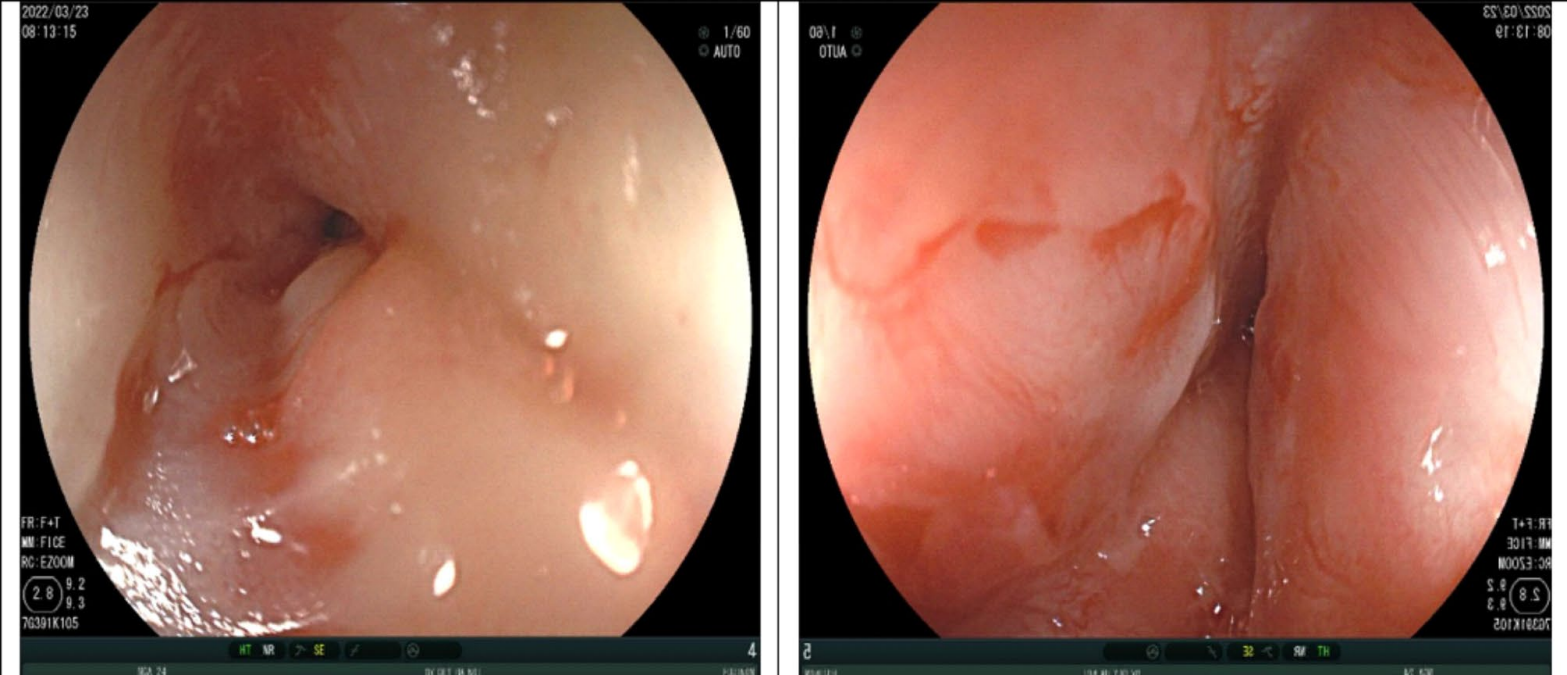
The upper endoscopic examination showed smooth submucosal swelling in the esophagus under 23 cm from the upper dental arch with a circumferential severe stricture which led to the endoscope does not passing

**Fig. 2:**
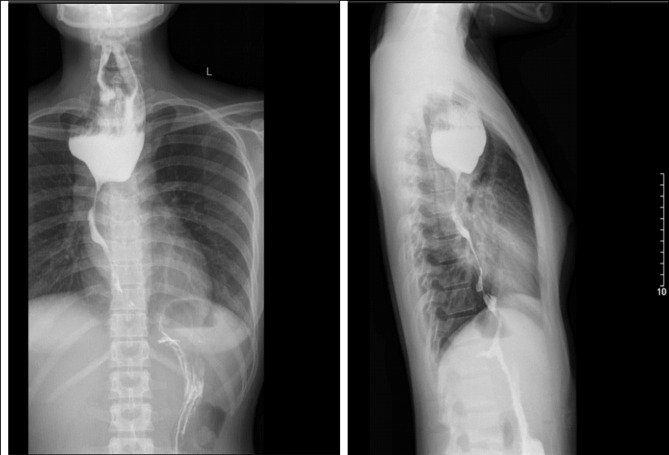
Barium swallow showed that the drug was circulating through the esophagus to the cardia but narrowing of the middle third of the esophagus in the 21 cm-long segment and stagnation in the upper segment

**Fig. 3:**
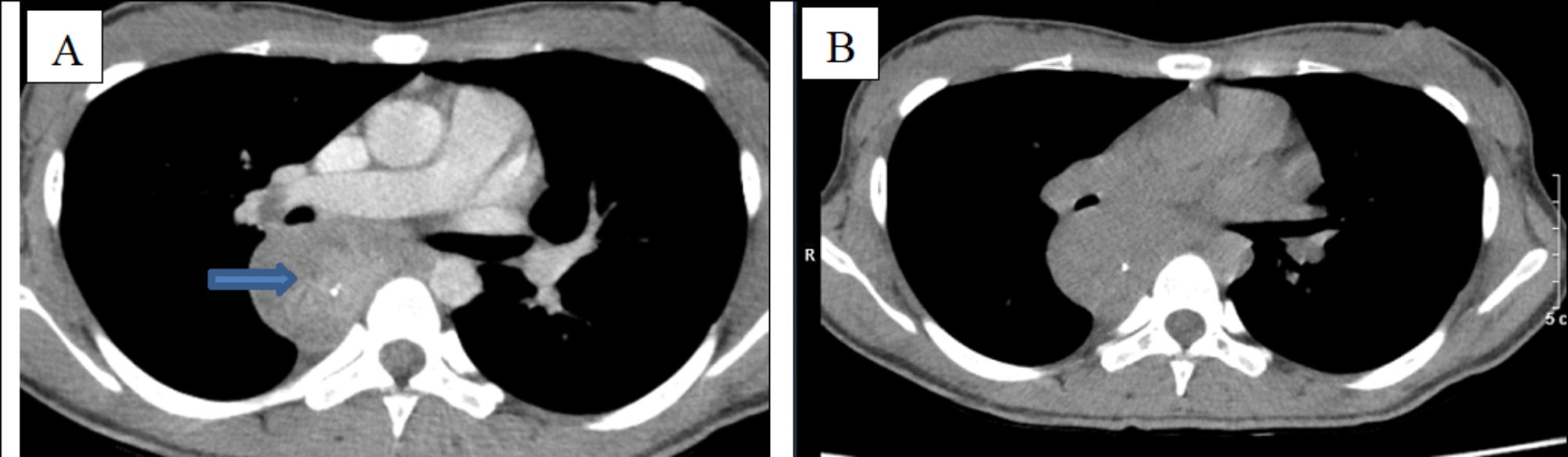
Tumor lesions of the esophagus have regular borders, clear boundaries with some calcified nodules (blue arrow), a pre-injection hypoattenuating lesion **(B)** and strong non-homogeneous enhancement after injection **(A)**, contact, push and cause slight narrowing of carina and left main bronchus, unknown invasion encroaching on the surrounding organs

A 24-year-old female patient has obstetric history of one time 6-months-premature born, one time of miscarriage, and diagnosed with a double uterus. The patient has showed signs of dysphagia increasing gradually and losing 10 kg in weight for 2 months. The patient has entered our hospital in severe malnutrition state of Body Mass Index (BMI) was 16.4 (height 150 cm, weight 37 kg), grade-3-dysphagia (can only drink water), no difficulty in breathing. The blood test results were total leukocytes count of 19.07 G/L, the rates of neutrophils and lymphocytes were 85.7% and 8.9% respectively, the serum albumin result was 32.2 g/l, high-sensitivity C-reactive protein (CRPhs) test was 20.78ng/dL, the levels of CEA and CA19-9 were 0.3 ng/mL and 17.6 U/ ml respectively, other tests were within normal limits. The upper endoscopic examination showed smooth submucosal swelling in the esophagus under 23 cm from the upper dental arch with a circumferential severe stricture which led to the endoscope does not passing, and the biopsy showed the pathological results were chronic inflammation and fibrosis (Fig. 1). Barium swallow showed that the drug was circulating through the esophagus to the cardia but narrowing of the middle third of the esophagus in the 21 cm-long segment and stagnation in the upper segment (Fig. 2). Computed tomography (CT) showed an irregular thickening of the esophageal middle and lower thirds segment around the circumference, the thickest part was 18 mm on the 10-cm-length, causing narrowing of the esophageal lumen, dilating the upper esophagus. Tumor lesions of the esophagus have regular borders, clear boundaries with some calcified nodules, strong non-homogeneous enhancement after injection, contact, push and cause slight narrowing of carina and left main bronchus, unknown invasion encroaching on the surrounding organs (Fig. 3). A biopsy of the esophageal tumor under the guidance of computed tomography was proceeded, and the pathological results were chronic sclerosing inflammation. The preoperative diagnosis in this case was smooth submucosal tumor led to esophageal stricture. Because of weight loss and malnutrition, the patient had preoperative nutritional consultation and intervention. Before surgery, the patient gained 5 kg compared to the time of admission, BMI before surgery was 18.7.

**Fig. 4:**
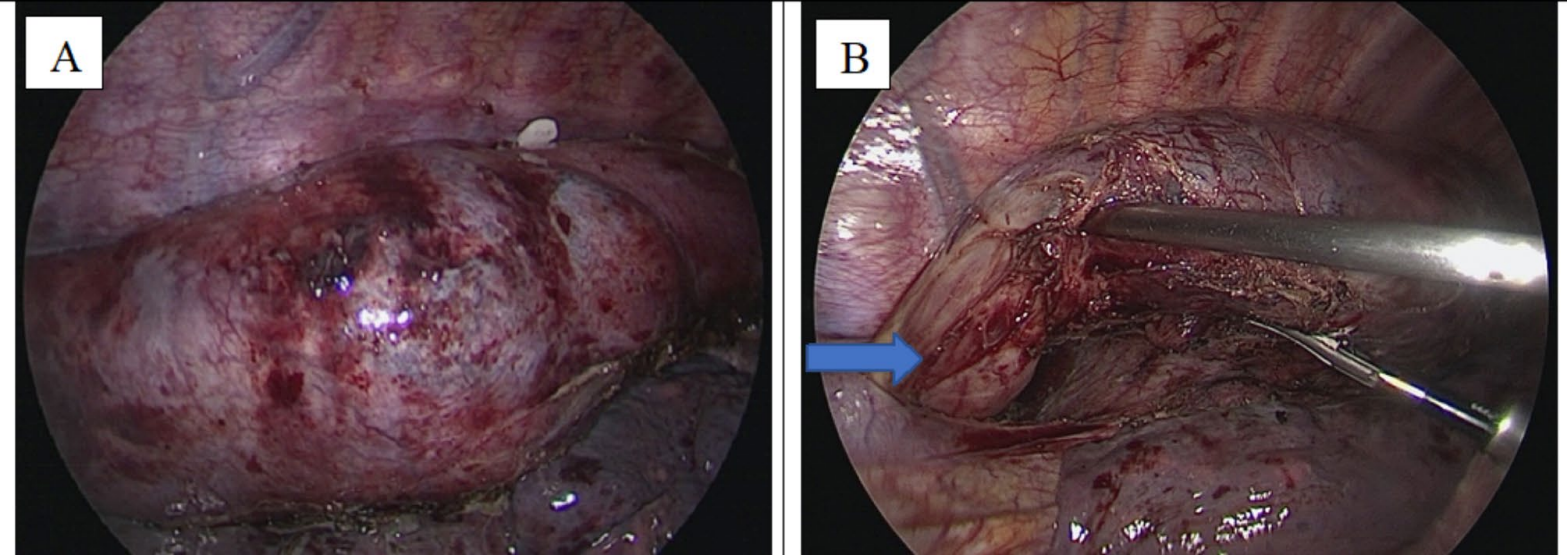
A large tumor, probably 12 × 5 cm in size, occupied the entire thoracic esophagus, and this mass was attached to the thoracic aorta, and the lymph nodes around the esophagus were enlarged and soft: **(A)** the tumor. **(B)** normal portion of esophagus (blue arrow)

**Fig. 5:**
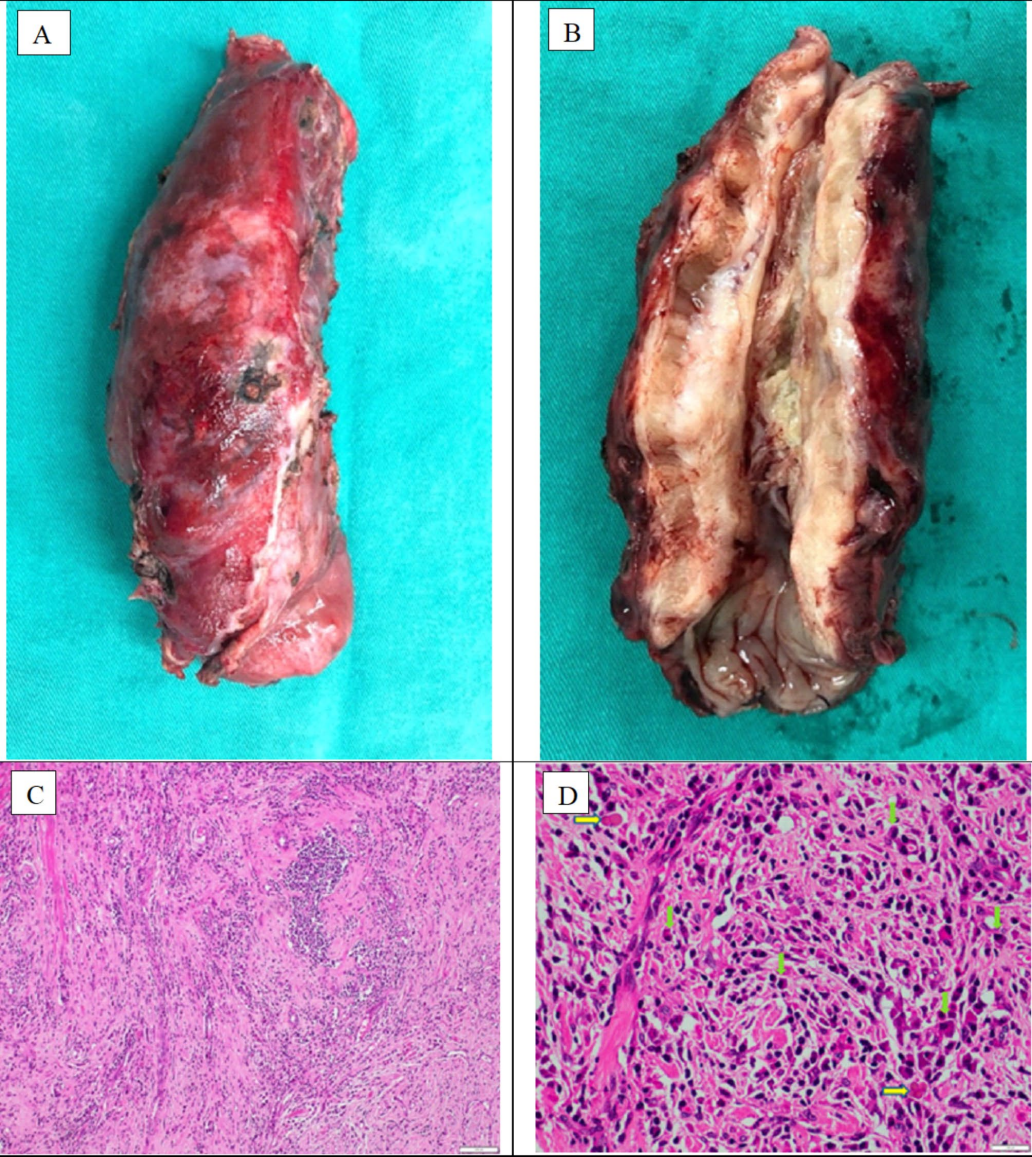
Macroscopic and microscopic characteristics of the lesion. **(A)** The tumor occupies most of the length of the thoracic esophagus. **(B)** The entire wall of the esophagus is a tumor caused circumferential severe stricture and a minor normal part of the esophagus (green arrow). **(C)** The layers of the esophageal wall show strong fibrous proliferation with infiltration of many inflammatory cells. **(D)** The inflammatory cell component consists mainly of plasma cells (blue arrows) with lymphocytes with occasionally eosinophilic Russell bodies (yellow arrowheads). (H&E x 400)

**Fig. 6:**
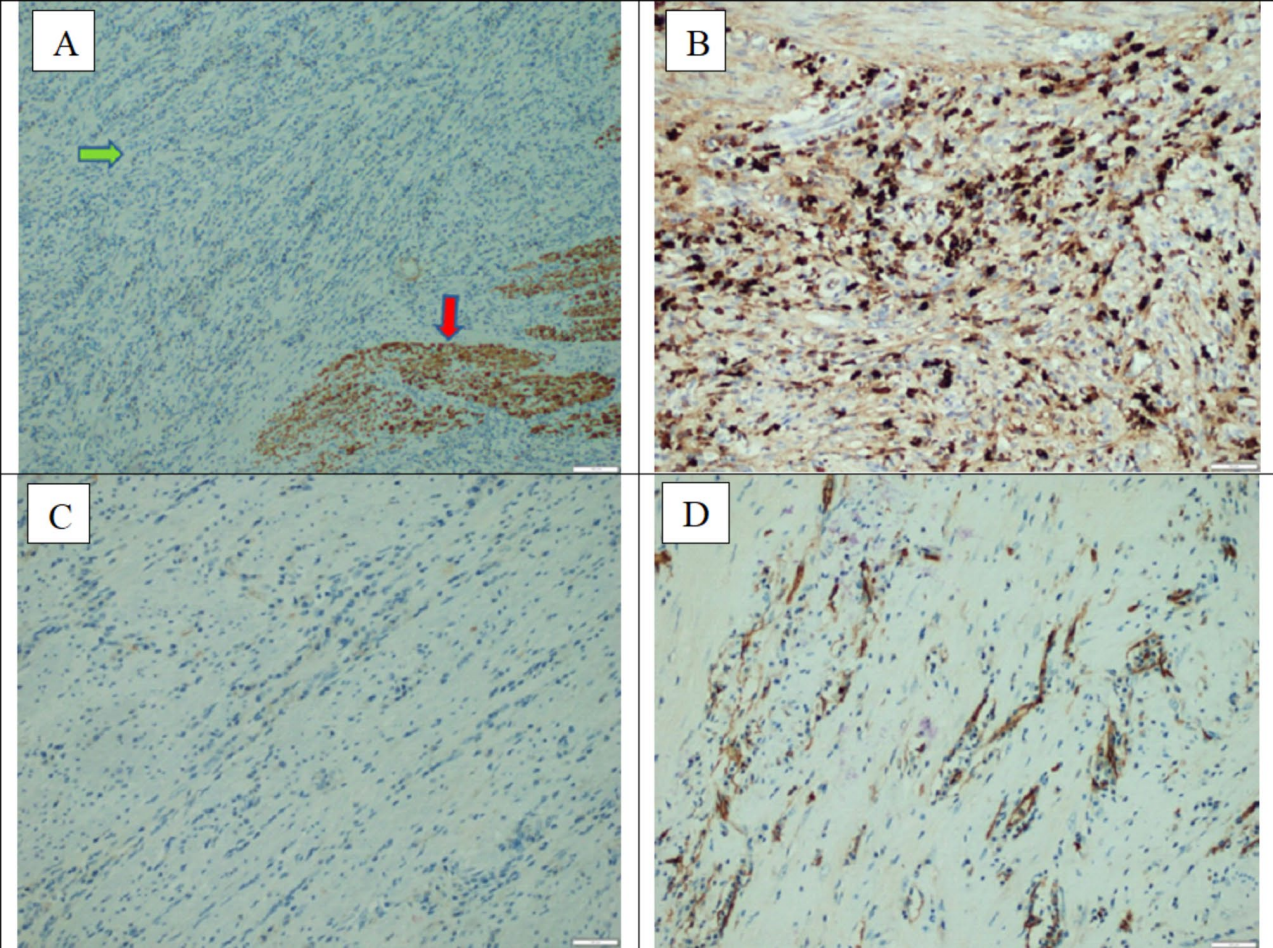
Immunohistochemical staining. **(A)** Desmin marker staining was positive only for smooth muscle fibers of the esophageal wall (red arrow), fibrous proliferative stromal area with inflammation was completely negative (blue arrow) (original magnification x 100). **(B)** An increase in the number of IgG4-positive plasma cells with the number about 120 IgG4(+) plasma cells /high power field (original magnification x 400). **(C)** The rhabdoid fibroblasts are negative for ALK1. **(D)** The rhabdoid fibroblasts are negative for CD34

An inter-department consultation of Gastroenterology - Radiology - Surgery - Pathology was proceeded. Due to the aggressive clinical symptoms and gross lesion characteristics, the patient underwent laparoscopic-thoracoscopic esophagectomy and reconstruction with a gastric tube. A large tumor, probably 12 × 5 cm in size, occupied the entire thoracic esophagus, and this mass was attached to the thoracic aorta, and the lymph nodes around the esophagus were enlarged and soft (Fig. 4). The patient underwent laparoscopic-thoracoscopic esophagectomy and reconstruction with a large gastric tube, made an anastomosis of the cervical gastro-esophageal tube. After surgery, the patient was stable and discharged after 9 days. 4 months after surgery, the patient gained 4 kg, eating normally. Histopathological examination showed that the squamous epithelium of the esophagus had a small, benign nucleus, the submucosal layer and the smooth muscle layer increased fibrous, with infiltrating many lymphocytes, plasma cells, and macrophages (Fig. 5). Immunohistochemical staining was negative for CD68, CD34, Desmin and ALK markers. However, there was an increase in the number of IgG4-positive plasma cells (Fig. 6). The final diagnosis was esophageal inflammatory pseudotumor.

## Discussion and conclusion

IPT or has been known as term IMT (inflammatory myofibroblastic tumor) are pseudo-sarcomatous lesions of the viscera and soft tissues which often occur in children and young adults, however, IMTs have the greater proliferation of myofbroblastic cells than IPTs, which represent more of inflammatory cells than IMTs [11, 14]. IPT in the esophagus is an extremely rare lesion and develops from the submucosa. Several reports suggest that esophageal IPT often has superficial mucosal ulcers [10]. Our case did not show mucosal ulceration on endoscopy. So far, the etiology and pathogenesis of IPT is not clear, it can be seen that some cases are related to Ebtein-barr virus, mycobacterium, immune-autoimmune condition, trauma, inflammation caused by surgery or patients with an abnormality in wound healing [5, 11, 15]. In our patient’s case, the disease progressed slowly and gradually, and no relevant cause was found. The characteristics in radiologic investigations of IPT are nonspecific, possibly because of the variable amount inflammation cellular infiltration and strong fibrous proliferation. On computed tomography, IPT is usually a pre-injection hypoattenuating lesion and a heterogeneous strong enhancement after injection, with calcified lesions in the tumor, which was found these features in our patient’s case [11, 16].

Depending on the location, size, and clinical presentation, esophageal IPT can be treated with endoscopic gastroesophageal resection, surgical excision, tumor resection, or esophagectomy [8, 16]. The choice of surgical excision or esophagectomy must be based on a comprehensive assessment and carefully considering radical resection ability, the recurrence risk and surgical complications. The method of local excision is usually performed in cases where the tumor is smaller than 2.5 cm and there is no evidence of tumor invasion of the muscle layer on endoscopic ultrasound, otherwise, esophagectomy is usually performed in cases of the tumor larger than 2.5 cm, or the tumor caused esophageal obstruction, or there is evidence of tumor invasion of the esophageal muscle layer [17]. With such treatment, the local recurrence rate is less than 10% [18]. In our case, the patient showed signs of choking, could only drink water, showed esophageal obstruction, 10 kg weight loss, on computed tomography of the esophagus there was a very large tumor, occupying most of the thoracic esophagus, so that we decided performing esophagectomy to completely removal the tumor.

Histopathologically, IPT contains acute and chronic inflammatory cells including lymphocytes, plasma cells, rhabdoid fibroblasts, and collagen (inflammatory response) [4, 11]. Several cases of IPT have been found IgG4 positive plasma cells which be associated with sclerosing disease, a systemic disease in which extensive infiltration of plasma cells, T cells, and various tissues is positive for IgG4 [11]. This condition manifests in autoimmune pancreatitis, sclerosing cholangitis, salivary gland inflammation, retroperitoneal fibrosis, tubulointerstitial nephritis, and interstitial pneumonia [19]. IgG4-associated IPT has been found in both patients with and without autoimmune pancreatitis, and in these case, systemic corticosteroids therapy was considered, especially in cases of incomplete resection [20, 21]. Our patient showed an increased number of IgG4-positive plasma cells (the number is about 120 IgG4(+) plasma cells per one HPF with original magnification x 400). IPT is distinguishable from lymphoma because IPT has both B and T cells, whereas lymphoma usually has only T cells or B cells [12]. In our case, the tumor was completely removed by esophagectomy, and the symptoms totally disappear in long-term follow-up, so systemic corticosteroids therapy was not considered.

### Conclusion

Inflammatory pseudotumor of the esophagus is a very rare benign lesion that be difficult to make a definitive diagnosis preoperatively but could led to aggressive clinical presentation. The gold standard of IPT diagnosis is histopathological examination of surgically removed specimens. Radical resection is still the most efficient treatment method, while systemic corticosteroids therapy was considered in case of immune-relation and incomplete resection.

## Data Availability

Data is available upon reasonable request and with permission of Hanoi Medical University Hospital.

## Abbreviations

ALK1: Anaplastic lymphoma kinase
CA 19 − 9: Carbohydrate Antigen 19 − 9
CD: Cluster of differentiation
CEA: Carcinoembryonic antigen
CT: Computed tomography
CRP: C-reactive protein
IgG4: Immunoglobulin G4
IMT: Inflammatory myofibroblastic tumor
IPT: Inflammatory pseudotumor
HPF: High power field
H&E: Hematoxylin and eosin
